# Meningiomas with different histological grade in the same patient: Case report

**DOI:** 10.1097/MD.0000000000009086

**Published:** 2017-12-15

**Authors:** Yang Liu, Da-Ping Song, Tong Wang

**Affiliations:** a Department of Neurosurgery; b Department of Pathology; c Department of Neurosurgery, The Third Hospital of MianYang, Sichuan, China.

**Keywords:** atypical meningioma, benign meningioma, different histological grade

## Abstract

**Rationale:**

Meningiomas are mostly regarded as benign tumors histologically,accounting for 13% to 26% of all primary intracranial tumors.It is testified that multiple meningiomas occur in <10% of cases.A case of concurrent grades I and II in the same patient in our hospital was described.

**Patient concern:**

A 66-year-old man who was experiencing headache and weakening in the left limbs, which gradually improved.Then, the myodynamia of left limb was weakening to level 3 and the muscular tension of left limbwas too strong for 1 year. Finally the man was admitted to our department of neurosurgery.

**Diagnoses:**

According the symptoms, signs and imaging data the patient. The 2 masses was diagnosed as the meningioma.Finally the histological examination showed the meningioma located in the right parietal lobe was diagnosed as fibrous meningioma,WHOgrade I, whereas meningioma reaching to the skull as atypical meningioma, WHO grade II.

**Interventions:**

The 2 masses including the invaded dura mater,parietal skull, and adjacent subcutaneous tissue were excised wholly In the process of surgery.

**Outcomes:**

There is no sign caused by recurrent tumor. within the half year.The physical of the patient is good

**Lessons:**

The patient with multicentric meningiomas should keep follow-up closely in case the meningiomas show the malignant characteristics.

## 1 Introduction

Generally speaking, meningiomas are classified by the 2016 World Health Organization (WHO) into grade I (benign), II (atypical), and III (anaplastic).^[1]^ Meningiomas are mostly regarded as benign tumors histologically, accounting for 13% to 26% of all primary intracranial tumors^[2]^; atypical meningiomas are testified approximately 20% to 35% of all meningiomas.^[3]^ It is testified that multiple meningiomas occur in <10% of cases.^[4]^ Moreover, and now it seems so far away, report about concurrent meningiomas in different grades is extremely rare.^[5]^ As follows, a case of concurrent grades I and II in the same patient in our hospital was described. And we got the permission of the Third Hospital Ethics Committee of MianYang to publish this case.

## 2 Case report

**Figure 1:**
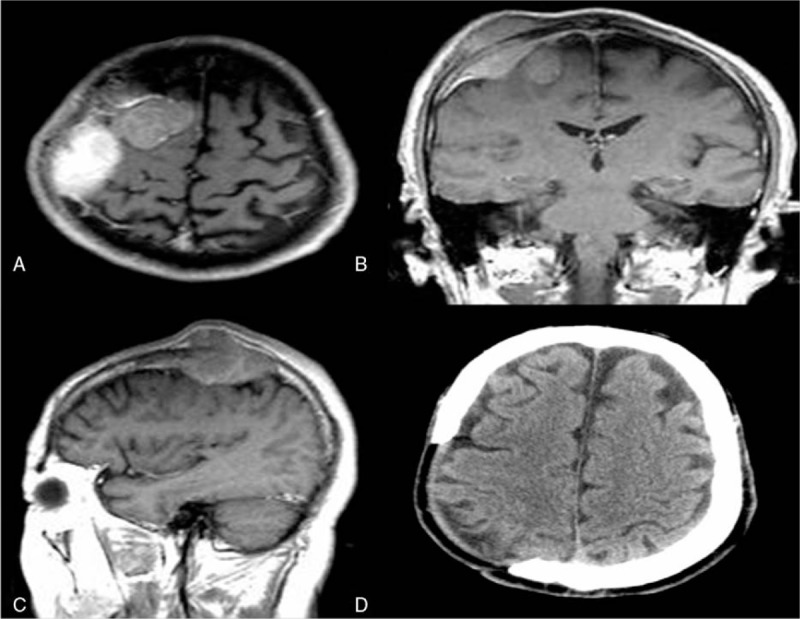
Preoperative axial (A), coronal (B), and sagittal (C) T1-weighted magnetic resonance images show 2 adjacent meningiomas in the right parietal lobe. Precontrast computed tomography scan taken on the fifth month after operation (D).

A 66-year-old man who was experiencing headache for 1 year was admitted to our department of neurosurgery. There was a scalp protrusion on the right parietal which measured 2 × 2 cm^2^. Patient had weakened in the left limbs, which gradually improved. Finally, the myodynamia of left limb was weakening to level 3 and the muscular tension of left limb was too strong. No computed tomography or magnetic resonance imaging (MRI) had been performed before. The patient had no obvious history other than headache. MRI showed a 3.9 × 3.6 × 3.8 cm^3^ mass lesion in the right parietal lobe. The mass was reaching to the skull without an enhanced dural tail extension (Fig. 1). Another 5.2 × 4.0 × 3.9 cm^3^ mass lesion was detected between the scalp and the right parietal lobe with heterogeneous signal (Fig. 1).

**Figure 2:**
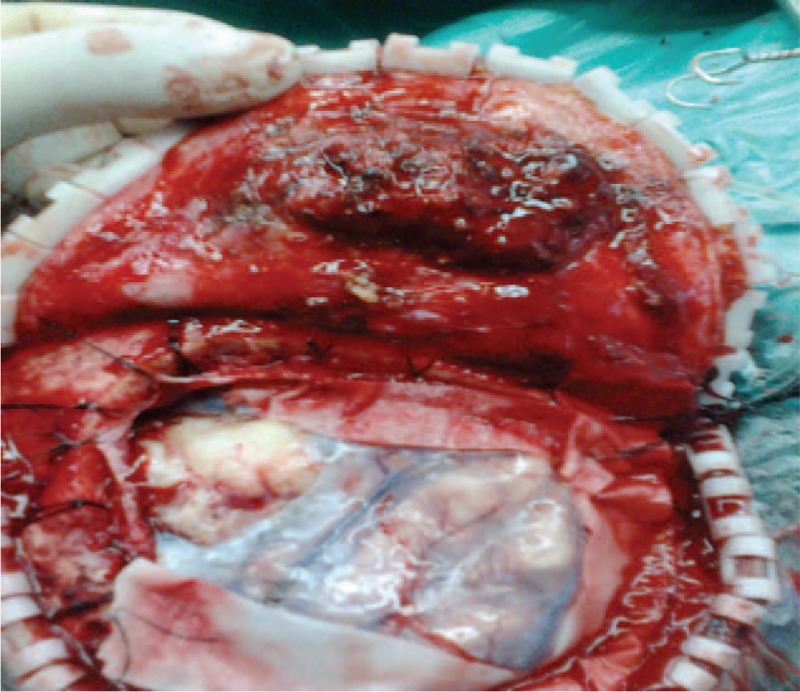
WHO I meningioma located between the right parietal lobe and cerebral pia mater; WHO II grade meningioma located between galea aponeurotica and cerebral pia mater.

**Figure 3:**
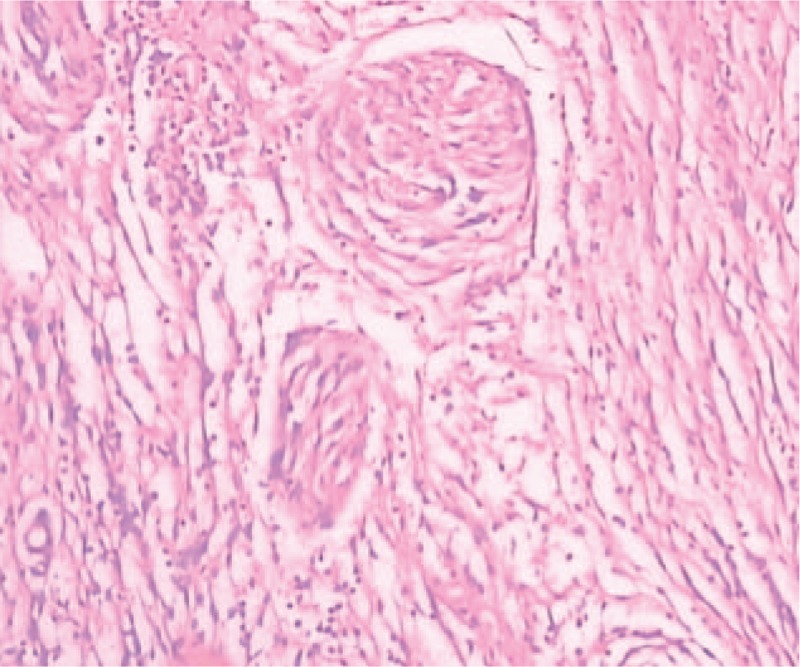
Histopathologic examination revealed a WHO I grade meningioma, hematoxylin, and eosin staining; original magnification ×200.

**Figure 4:**
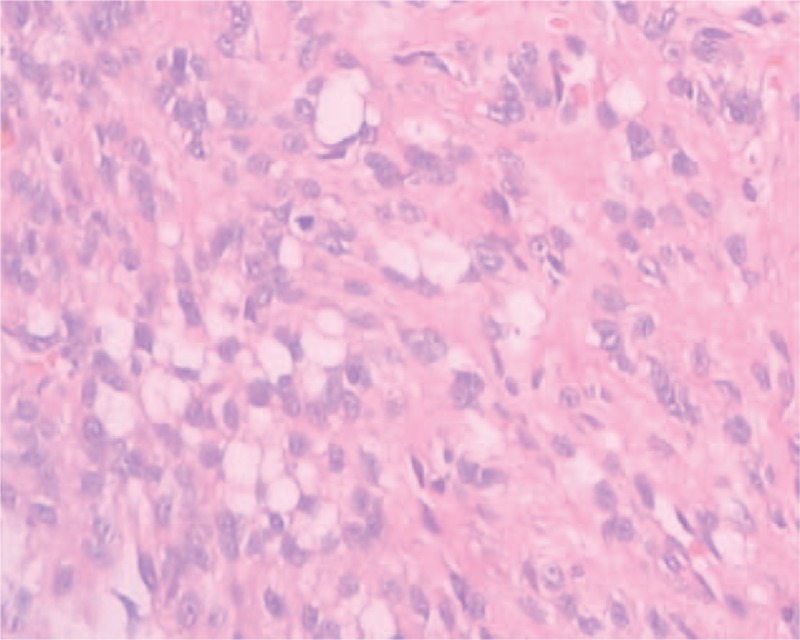
Histopathologic examination revealed a WHO II grade meningioma, hematoxylin, and eosin staining; original magnification ×200 (5 mitoses in 1 high-power field).

In the process of surgery, indirect connections were found in 2 lesions. Outside of meningioma, there was a rich vascular supply invading skull, dura mater, and galea aponeurotica. Inside of meningioma, without rich blood flow located in the right parietal lobe, meanwhile, cerebral pia mater and brain tissue were not destroyed. The 2 masses including the invaded dura mater, parietal skull, and adjacent subcutaneous tissue were excised wholly. Resected tumors were performed histological examination by 2 independent pathological experts. The meningioma located in the right parietal lobe was diagnosed as fibrous meningioma, WHO grade I, whereas meningioma reaching to the skull as atypical meningioma, WHO grade II. In atypical meningioma, Ki-67 staining index is approximately 20%. Supplementary treatments such as radiotherapy and chemotherapy were rejected. This patient was discharged and close follow-up was recommended (Figs. 2–4).

## 3 Discussion

The annual occurrence of meningiomas is estimated to 2 to 7/100,000/year for women and 1 to 5/100,000/year for men, and the incidence rate adjusted on the world wide population is 3.634/100,000/.^[6–8]^ The incidence of meningiomas is increasing over time, particularly in the elderly, from which such increase is resulted cranial imaging, better imaging facilities, and aging populations.^[6,9]^ In the past, it was common to see that the incidence rate of atypical and anaplastic meningiomas in men is higher, which is possibly relevant to the higher proliferation indices that discovered in meningiomas of male patients.^[10]^ In recent years, The greater frequency of such tumors among adult females is well known on the contrary, which is caused from reproductive and hormonal factors.^[11]^ A majority of meningiomas arise from the arachnoid cap cells are histological benign, including fibrous meningioma, whereas some meningiomas present malignancy signs, such as marked vascularity, loss of organoid structure, mitotic figures, nuclear pleomorphism, prominent nucleoli, focal necrosis, or infiltration to the adjacent brain.^[12,13]^ Ki-67 staining index, a predictors for recurrence, was varied from 3.7% to 9% in most cases of atypical meningioma,^[14,15]^ but it is also reported that repeated recurrences in spite of the initial KI-67 staining index is 0.4%.^[15]^ What's more, compared the previous data with 20% of KI-67 staining index in our case, it ulteriorly proves that KI-67 staining index is only a reference index predicting recurrence.

In general, the cases that meningiomas are benign but with multicentric patterns have been increased in different series than previous.^[16,17]^ Multiple meningiomas account for 1% to 10% of meningiomas.^[18]^ A widely accepted hypothesis of multicentric meningioma indicates that tumoral cell clone spread through cerebrospinal fluid and hematogenous expansion. It's rare to find different sporadic patterns in 1 patient. In previous literatures, atypical and psammomatous meningiomas^[19]^ or concurrent fibrous and atypical meningiomas^[20]^ have been occasionally found in different intracranial locations. Coincidentally, in our study concurrent benign meningioma and atypical meningiomas are identified by histopathology with immunohistochemical analysis. The results above demonstrated the concurrent development of independent tumors.^[21,16]^ The pathological classification contribute to different treatment. WHO grade I meningioma usually require total excision of the tumor and invaded dura mater. Supplementary treatment including radiotherapy and chemotherapy is indispensable because WHO grade II meningiomas have malignant character. In follow-up period, if necessary, the secondary surgery may be carried out. In our study, the tumors, invaded dura mater, and skull have been excised totally (Simpson I) in order as much as possible to avoid recurrence. Tumors may be not be stopped from growing back due to the lack of complementary therapies; therefore, this patient is to be requested to keep follow-up closely.

## 4 Conclusion

In brief, we report an unusual case of a 66-year-old patient with 2 meningiomas concurrent. Indirect connection, different vascular supply, and invasiveness are characteristics of our case. Finally, we diagnosed this case as fibrous meningioma WHO grade I, and atypical meningioma WHO grade II. Multiple meningioma patterns have been increasing gradually, but in different histological grades is extremely rare. We accumulate much experience in deal with this case with different histological grades. The patient with multicentric meningiomas should keep follow-up closely in case the meningiomas show the malignant characteristics.
